# Tick Paralysis: Solving an Enigma

**DOI:** 10.3390/vetsci5020053

**Published:** 2018-05-14

**Authors:** Ronel Pienaar, Albert W. H. Neitz, Ben J. Mans

**Affiliations:** 1 Epidemiology, Parasites and Vectors, Agricultural Research Council–Onderstepoort Veterinary Research, Onderstepoort, Pretoria 0110, South Africa; 2 Department of Veterinary Tropical Diseases, Faculty of Veterinary Science, University of Pretoria, Onderstepoort, Pretoria 0110, South Africa; 3 Division of Biochemistry, University of Pretoria, Hatfield, Pretoria 0028, South Africa; albert.neitz@up.ac.za; 4 Department of Life and Consumer Sciences, University of South Africa, Florida, Johannesburg 1710, South Africa

**Keywords:** blood-feeding, tick evolution, tick paralysis, tick toxicoses, venom, toxin

## Abstract

In comparison to other arachnids, ticks are major vectors of disease, but less than 8% of the known species are capable of inducing paralysis, as compared to the ~99–100% arachnids that belong to venomous classes. When considering the potential monophyly of venomous Arachnida, this review reflects on the implications regarding the classification of ticks as venomous animals and the possible origin of toxins. The origin of tick toxins is compared with scorpion and spider toxins and venoms based on their significance, functionality, and structure in the search to find homologous venomous characters. Phenotypic evaluation of paralysis, as caused by different ticks, demonstrated the need for expansion on existing molecular data of pure isolated tick toxins because of differences and discrepancies in available data. The use of in-vivo, in-vitro, and in-silico assays for the purification and characterization of paralysis toxins were critically considered, in view of what may be considered to be a paralysis toxin. Purified toxins should exhibit physiologically relevant activity to distinguish them from other tick-derived proteins. A reductionist approach to identify defined tick proteins will remain as paramount in the search for defined anti-paralysis vaccines.

## 1. Introduction

Ticks are obligatory hematophagous organisms with life stages that may share different niches or feed on different hosts. The effect that they have on their hosts is traceable through direct and indirect damage that is associated with feeding and through the enormous financial and economic burden that they impose on successful stock farming practices [1,2]. They are classified as arachnids (Arachnida) of the subclass Acari, Order Ixodida, and superfamily Ixodoidea [3]. Three families exist: the Ixodidae (hard ticks), the Argasidae (soft ticks), and the monotypic Nuttalliellidae [4]. Besides being vectors of disease, some ticks are able to induce various forms of toxicoses that include paralysis [5,6,7,8,9,10] and general toxicoses. Toxicosis exhibit in diverse forms, as described by Gothe and Neitz [11,12] and are associated with tick feeding. They include: sand tampan toxicosis that is caused by the nymphs and adults of *Ornithodoros savignyi*, [13], sweating sickness, Mhlosinga and Magudu, and necrotic stomatitis nephrosis syndrome that is caused by *Hyalomma truncatum* [14,15], a vague report on toxicosis from *Rhipicephalus microplus* [16], toxicosis from *Dermacentor marginatus* [11,17], *Rhipicephalus appendiculatus* [18], *Ixodes redikorzevi* mainly affecting man [11,19], and toxicosis from *Ornithodoros gurneyi* [20].

Tick paralysis, on the other hand, is considered to be the most important of the toxic phenomena associated with feeding ticks, especially with regards to veterinary and human medicine. This is evident in the new rapidly emerging field of Internet-based surveillance built on Internet search queries for “tick paralysis” that enable the early detection of disease or exposure during high-risk periods [21]. Of the 900 odd tick species, fifty-nine ixodid and fourteen argasid species are implicated in tick paralysis [10], with many other records being uncertain and incomplete. The functional significance of why ticks produce toxins are still unclear. It is generally accepted that tick paralysis is caused by neurotoxins produced in the salivary glands and is secreted into the host during a specific time in the course of feeding by the female or immature stages of the species. Analysis of the role of tick saliva in host interaction has rapidly expanded over the past decade with the explosion of “-omics-technologies” [22,23,24,25,26,27,28,29]. This confirmed and expanded on the known diversity of molecules that tick saliva has in its arsenal of anti-hemostatic, anti-platelet, anti-coagulant, and vasodilatory compounds [27,30,31]. The role of tick saliva in pathogen transmission by means of bioactive molecules in saliva-assisted transmission has also become more prominent [27,29]. Recently, the identification, molecular and structural characterization of the only characterized paralysis causing toxin, holocyclotoxin from *Ixodes holocyclus*, has advanced our insight into tick paralysis significantly [32,33,34,35]. However, the characterization and the mechanism of most of the tick toxins that are secreted by ticks implicated in paralysis are lacking and need to be addressed to attribute functional significance to tick toxins, as compared to other known arachnid toxins. The majority of the known arachnid toxins are lineage specific, secretory proteins with characteristics that are not necessarily shared between lineages [36]. For the majority of tick paralysis toxins, no sequence data exists to define their molecular identity and it is unknown whether tick toxins are evolutionarily related among ticks, or to other arachnid toxins. 

**Figure 1:**
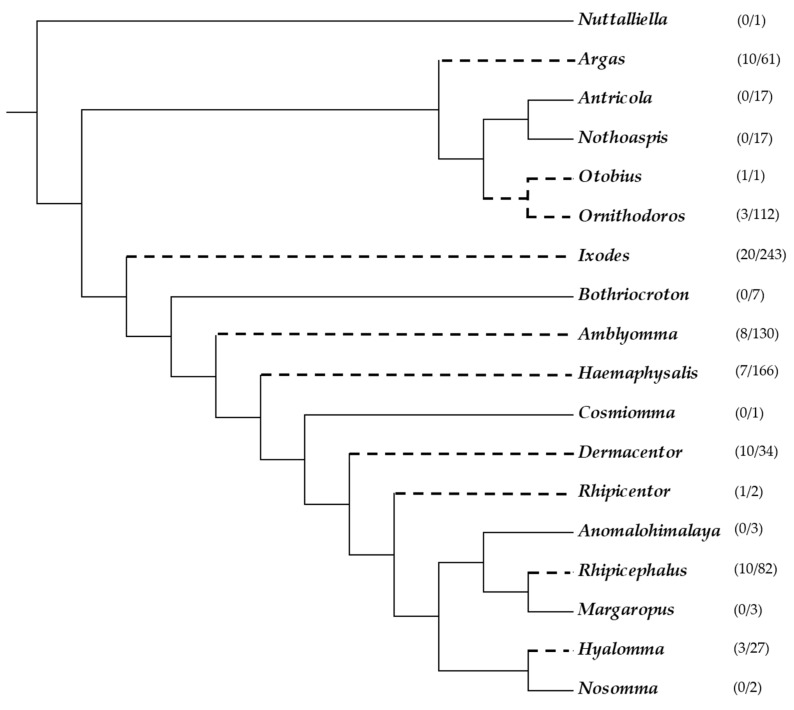
Phylogenetic relationships of the 73 ixodid and argasid tick species that were implicated in paralysis adapted from [36]. Broken branch lines indicate genera that are implicated in paralysis. Numbers in brackets indicate the number of species implicated in paralysis or paresis as updated [10], followed by the total number of species [4].

The latest list of ticks that are able to cause paralysis or paresis consist of 73 species (Figure 1), of which 14 are from the family Argasidae, including 10 species from the genus *Argas*, 3 species from the genus *Ornithodoros* and *Otobius megnini*. The family Ixodidae is represented by 59 species, including eight from the genus *Amblyomma*, 10 from the genus *Dermacentor*, seven from the genus *Haemaphysalis*, three from the genus *Hyalomma*, 20 from the genus *Ixodes*, 10 from the genus *Rhipicephalus*, and one species of *Rhipicentor* [10]. Paralysis species, therefore, comprise less than 8% of all known tick species and this gives an interesting comparison to other arachnids that are considered to be venomous, such as spiders (~99% venomous from ~47,000 species) or scorpions (100% venomous from ~2300 species) [37,38]. Of these, only *Argas walkerae* (South Africa), *I. holocyclus* (Australia), *Ixodes rubicundus* (South Africa), *Dermacentor andersoni*, *Dermacentor variabilis* (North America), and *Rhipicephalus evertsi evertsi* (Africa), is considered as species that cause significant paralysis in host species [7,39], making the number of species for which frequent paralysis is observed, less than 1% of all tick species.

## 2. The Enigma: Arachnida as a Monophyletic Venomous Group: Implications for the Origins of Tick Toxins

The Arachnida is a monophyletic group composed of various venomous taxa, suggesting as the most parsimonious explanation, that venom evolved once in this group. However, it is recognized that venom from various arachnid taxa evolved independently [23,40]. Even so, based on the relationship of ticks to other venomous arachnids, it has been proposed that ticks should be classified as venomous, with the implication that the ancestral tick lineage was venomous as well [41]. The null hypothesis would therefore be that tick paralysis toxins share an evolutionary relationship among ticks (i.e., tick paralysis toxins are homologous), but also with other arachnids. While this argument seems to be the most parsimonious regarding the origin of tick paralysis toxins, it was indicated that the relationship of ticks and other venomous arachnids are not clear, and that the majority of arachnid lineages, except for scorpions and spiders, are not venomous [36]. Total evidence data by Klompen, J.S.H. et al. [42] demonstrated that the sister group of the Ixodida is the Holothyrida, which is related to the Mesostigmata and Opilioacarida. Holothyrids and other parasitiform mites do not have oral toxins, which make the possibility that ticks acquired toxins from their ancestors remote [36]. As such, various molecular characteristics of paralysis causing toxins suggest that toxin secretion evolved independently on multiple occasions, while systematic relationships suggest that the ancestral tick lineage was non-predatory or non-parasitic, and therefore non-venomous [10,31,36].

Comparison of salivary gland proteins from soft and hard ticks indicate that few orthologs exist that function at the tick-host interface, suggesting that the different tick families utilize different strategies in blood meal acquisition, and evolved most of their repertoires after divergence of the main tick families [36]. Using the same rationale, paralysis toxins from different tick families may also not be homologous and may have evolved independently [33,40,43]. The current review considers the evidence for a monophyletic origin of tick toxins and possible strategies to determine this.

### 2.1. Are Ticks Venomous Animals?

Twenty-first century research assigned ticks (and other arthropod lineages) the status of being venomous animals based on findings that reviewed convergent recruited proteins in the venom of various animals, and based on these grounds, ticks have been classified as venomous animals [41]. This study derived from a larger previous study that showed that many protein families and folds are shared among venomous animals [40]. The broad perspective that is provided in the latter study does not allow for the assessment of whether ticks and other arachnids shared a common venomous ancestor. However, a central message of this latter study was the convergence that was showed by different venomous lineages to utilize the same folds even though toxin functions evolved independently. Analysis of protein family presence in secretions indicated that ticks share five protein families with spiders and scorpions, respectively, but eight families with insects and reptiles [40]. Conversely, no neurotoxin protein families were indicated for ticks and no factors that target hemostasis indicated for scorpions or spiders. The “venomous” status was based on general characteristics of venoms from diverse organisms, namely: disulfide-bond rich, secretory proteins that belong to the same secretory protein families and exhibit similar functions. Venoms act rapidly and their diversity is due to gene duplication and the hyper-mutation of surface exposed residues. Production is located in specialized glands in the venomous animal and the delivery of venom to the host/prey is through a wound that leads to the disruption of physiological and biochemical processes to facilitate feeding or as a defense response [40].

Based on the definition above, all of the blood-feeding arthropods are venomous, including such relatively innocuous culprits, such as bed bugs, midges, mosquitoes, and tsetse flies. It should be noted though, that the inclusion of blood feeding arthropods was omitted in more recent reviews on the topic [43], and the clarification on the “function debate” of venom does not explain the presence of a trait or how it came to be [44]. Nonetheless, the venomous definition as it stands obscures the line between “toxic” and “benign” blood-feeding arthropods.

Secretions intrinsically contain secretory proteins that is disulphide-bond rich [45]. The recruitment of protein family members through gene duplication, are also no surprise since this forms the basis of the evolution of new functions from existing protein family members [46]. Mutation of surface exposed residues may be due to the relaxation of negative selection in gene duplicates, since few of these residues may be involved in function or structural maintenance, and not necessarily due to positive selection (hyper-mutation) [47]. 

Most arthropods have specialized glands (albeit salivary glands, prosomal glands, or venom glands, as discussed in Section 2.2) that produce secretory proteins, and blood-feeding by default imply that a wound of some sort will be inflicted on the host. All blood-feeding arthropods secrete bioactive components during feeding to enable the modulation of host defenses, in order to obtain a blood-meal. While these generalizations can hold for venomous organisms, they will also hold for any organism that secrete proteins during feeding. Defining ticks as venomous animals using a frame of reference that do not consider their entire biology may be premature. Similarly, attempting to postulate a common venomous ancestor for the greater arachnid class, without sufficient data on toxins from ticks that are capable of causing paralysis, to enable the assessment of their common ancestry to other arachnid toxins will also be premature. On the other hand, the composition of arachnid toxins is relatively well studied and comparative approaches may shed light on the relationships between various venomous organisms.

### 2.2. Do Arachnid and Tick Toxins Share a Common Ancestral History?

Elucidating the origin of tick toxins is controversial. It was suggested that the ancestral tick lineage was rooted in a venomous arachnid lineage, which includes spiders and scorpions, and might explain the different forms of tick toxicoses and paralysis observed today [40,41]. In order to elucidate the biological significance or search for the origins of tick toxins, a better understanding of the relationship and origin between Acari, Acariformes, and Parasitiformes with other arachnids is needed before character traits can be compared between arachnid lineages. However, the uncertainty pertaining to the sister group of the Parasitiformes, lack of molecular data on all of the toxins and the notion that toxins evolved independently in other arachnid lineages, make the search for toxin origins difficult. Mans, B.J. et al. [36] investigated the Ixodida and their relationships to other arachnids and considered the four possibilities to the sister group of the Acari as the Ricinulei, Palpigradi and Pychnogida, or as being unresolved. Closer relationships to spiders or scorpions has also been proposed. The sister group to ticks are the Holothyrida [42], which is a group of free-living mites living off body fluids of dead organisms. This would suggest that ticks shared the scavenging feeding habits of holothyrids before the adaption to a haematophagous lifestyle [48]. There is, therefore, a possibility that ticks were scavengers rather than predators with no functionality for toxins in salivary gland secretions. Thus, the origin of paralysis toxins seems to be intricately linked to the evolution of blood feeding and the ability of the tick to modulate the host’s immune system. 

The question remains, why are so many arachnids venomous? It may be postulated that the ancestral arachnid lineages were primarily predatory or parasitic. The selective drive for the evolution of toxins would have been to be more efficient at predation or parasitism. This may explain the independent evolution of venomous lineages in the Arachnida, since the driver would have been the improvement of efficiency as predators or parasites, but not primarily as venomous organisms. Independent origins of venoms may also be traced to the organs of origin and delivery systems. Spiders possess venom glands located in the basal segment of the chelicerae (mygalomorph spiders) or in their prosoma (araneomorph spiders), and these glands have been shown to be an ancestral trait [49]. Venom glands from the oldest spider groups (Mesothelae) are small and are considered to contribute little to predation success when compared to their well-developed chelicerae, suggesting that ancestral spider lineages were less venomous than the derived araneomorph lineages [50]. The use of venom (chemical attack) vs. chelicerae/fangs only (physical attack) to subdue prey depends on the prey to predator ratio, allowing for spiders to not only rely on being venomous alone. Ancestral spider lineages are therefore less venomous than derived lineages, with the implication that the venomous nature of spiders increased in derived lineages and did not necessarily derive from extremely venomous ancestors. 

Morphologically, spider venom glands are long and cylindrical, and are surrounded by a muscle layer that contract to secrete the venom. Venom gland cells are composed of a sheath of columnar cells that forms a lobe around a central lumen. This ultrastructural arrangement is similar to that of salivary glands from the Diptera [51]. Scorpions, on the other hand, possess paired venom glands that are located in their telson (stinger) at the tip of their tails. A thick muscular layer surrounds the glands and the cells are columnar organized around a central lumen [52]. Salivary glands from ticks are grape-like acini that are composed of multiple pyramidal cell types, and is unique in their morphological structure even compared to the prosomal glands of other Parasitiformes [53]. No evidence of structural homology exists with spiders or scorpions at this level, suggesting that molecules that were derived from these organs would also be non-homologous. 

With regard to delivery systems, spiders possess fangs (derived chelicerae) with channels into which the venom glands empty their contents, and inject them into the host after contraction of the muscles that surrounds the venom glands [50]. Scorpions, on the other hand, possess the telson and the stinger that sits at the end of their tails, to which the venom gland is connected [54]. The hypostome of ticks are a unique adaptation for blood-feeding that derive from the capitulum, that with the celicerae cuts into the host dermis and anchor the tick during feeding [55]. The chelicerae in this case fulfill the much more general function that is normally associated with chelicerae that is to cut into food sources. The delivery systems of scorpions, spiders and ticks are therefore also non-homologous and suggest that they evolved independently and specifically for their respective lifestyles.

## 3. Toxins and Venomous Organisms

### 3.1. Significance of Paralysis Toxins for Ticks

The reason why ticks need toxins, or produce them for that matter, is unclear, but the function of toxins in other arthropods are obvious, as in the case of spiders and scorpions, which utilize their toxins in defense and predation [56]. In the case of ticks, there are several possible reasons why ticks may possess paralysis toxins. It was proposed that paralysis might be a vestigial function that is left over from before the evolution of a parasitic lifestyle [57]. Some functional significance might include the impairment of host mobility and the grooming and stimulation of host seeking behavior due to an increased respiratory rate as a symptom of paralysis. This would lead to the increase of carbon dioxide release, which in turns, serve as cue to attract other ticks to the host [10,31]. Toxins might act as local analgesics, feeding stimulants, or play a role in the prevention of coagulation [57]. A protein synthesis regulatory role was suggested for *R. evertsi evertsi* after immune-localization to the chromatin in the nuclei of type II acini and theoretically the “b” cells [58,59]. 

While the above mentioned functions for toxins could be valid reasons for maintaining toxins as part of an organism’s biology, ticks in general do not use toxins as extensively as other arachnids. Most ticks do not paralyze their hosts (only 73 species from ~900 are implicated in paralysis) [10], and not all strains of the same species of ticks might have the ability to cause paralysis [60,61,62]. As such, the impairment of host grooming might be more important in the initial stages of attachment and establishment of the feeding site, through the secretion of molecules that act as analgesics, immune-modulators and anti-hemostatic agents to prevent pain and itching [53]. Paralysis toxins are, however, generally secreted just before the rapid engorgement phase (3–7 days), when the feeding site is well established. Ensuring feeding success by feeding on an immobilized host seems to be improbable, since most animals that carry ticks are relatively mobile if they are not terminally ill or immunosuppressed. Immobilization might even have a negative impact on the tick population should the host die due to predation or starvation before ticks have fed to repletion. The functions of analgesic, feeding stimulant, and to inhibit coagulation are notable adaptations in their own right. However, these and other functions that are involved at the tick-host interface has little to do with the paralysis phenotype. If paralysis toxins perform these functions, paralysis would be a secondary add-on effect not linked with the primary function. The functional role that tick paralysis toxins might play, as such, remain obscure.

### 3.2. What Is a Toxin?

A toxin (from Greek: *toxikon*) is described as a poisonous substance (molecules, peptides, or proteins) that is produced by living cells or organisms, which are capable of causing disease on contact or through absorption by tissues by means of interaction with macromolecules (enzymes or receptors), thus toxins need a target (receptor) to elicit their function. They are characterized by antigenicity in certain animals, not all display neurological symptoms affect the cardiovascular system, or are located in saliva. Generally, they are mostly associated with hypersensitivity reactions to component(s) at the feeding site in the host tissue, will cause a display of neurological symptoms that are characterized by nervous incoordination or affect the cardiovascular system. Tick salivary gland proteins have physiological function/s at the tick-host feeding interface and may be involved in evading the host’s immune system by neutralizing neutrophils, inhibiting inflammation, preventing blood clotting by preventing platelet aggregation, and insuring vasodilation [27,62,63]. Proteases, protease inhibitors, hyaluronidases, anticoagulants platelet aggregation inhibitors, and other hemolytic agents could also cause hypersensitivity reactions at the feeding site on the host [64]. These molecules typically do not cause an ascending paralysis, and have been identified in whole tick extracts, salivary gland secretions, salivary gland extracts and tick eggs [8,64]. Tick toxicoses, with clinical outcomes other than paralysis, have been associated with tick bites of *O. savignyi*, causing sand tampan toxicosis, *H. truncatum* toxicosis, toxicosis by *R. microplus*, *R. appendiculatus*, *D. marginatus*, *I. redikorzevi*, and *O. gurneyi* [11,12]. Other salivary gland proteins may be deemed toxic just because of a systemic reaction in the host, such as the inhibition of proteases important for host-specific physiological functions. However, not all salivary gland secretions can be regarded as toxic, as most are benign to the host.

Venom, on the other hand, is a mixture of toxins that is actively secreted by animals through bites or stings, mainly for predation or defense. The mechanism of activity of these venoms is either one or a combination of neurotoxins that may be involved in:

(1) Inhibition of ion, sodium, potassium, chloride, and/or calcium channels and synaptic vesicle release;
(2) Receptor agonists and antagonists;
(3) Cytoskeleton interference;
(4) Calcium-mediated cytotoxicity or; and,
(5) Neurotoxins with multiple effects.

Some of the proteins secreted by ticks share striking similarities to the protein compositions of conventional venomous secretions (venom). Even so, the molecules that target host receptors differ markedly in venomous arachnids. An appreciation can be gained through the extensive research that has been performed on toxins and venom transcriptomes for scorpions and spiders.

### 3.3. Scorpion Toxins and Venoms

**Figure 2:**
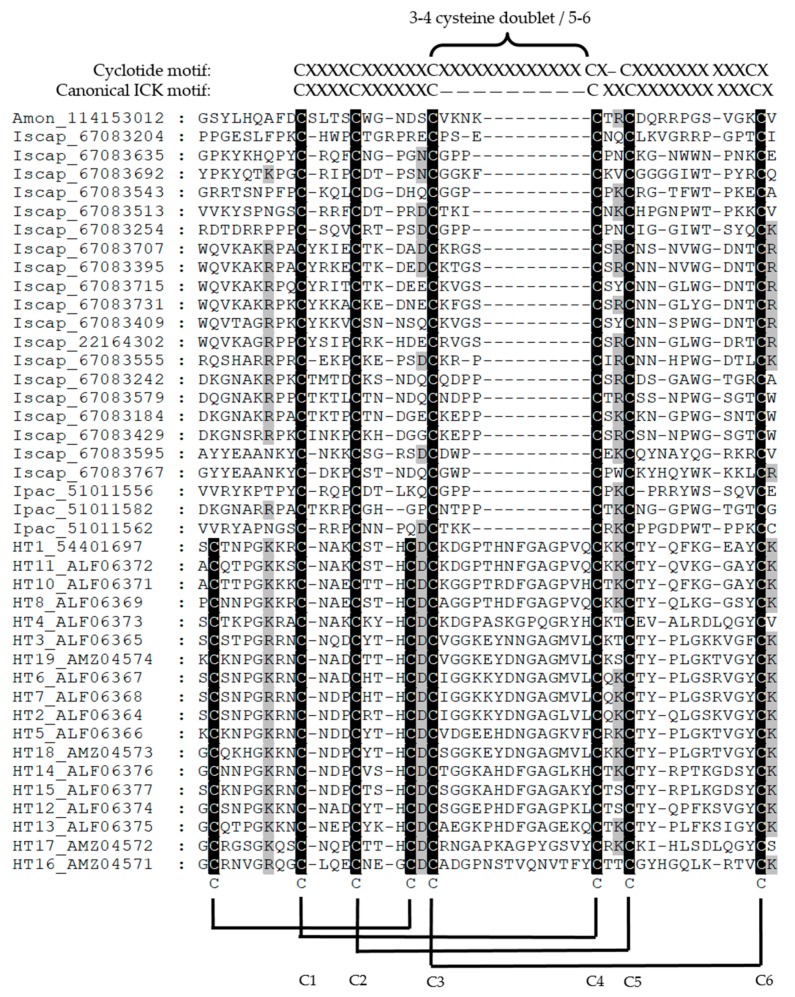
Alignment of the *Ixodes holocyclus* holocyclotoxins (HT) with members of the *Ixodes scapularis* (Iscap) *and Ixodes pacificus* (Ipac) 5.3 kDa family as well as a member from *Argas monolakensis* (Amon) followed by their corresponding Genbank accession numbers. Conserved residues are shaded in black and disulphide bonds from holocyclotoxin are indicated. The cysteine patterns of the canonical inhibitory cysteine knot (ICK) motif and the cyclotide variant pattern and the 3/4 cysteine doublet are indicated (cysteine 5–6 in holocyclotoxin). The cysteine number for the canonical ICK motif is indicated below the disulphide bonds.

Scorpion venoms are rich in small disulphide rich peptides [65]. The cytotoxic peptides comprise three families considered to have evolved from a common ancestor, and they include the bradykinin potentiating peptide family, the NDBP 5 linear family, and the short cationic antimicrobial peptide family [65]. These toxins cause cell haemolysis and death. They are not considered to be abundant in tick sialomes. The cysteine stabilized αβ scaffold (CSαβ) comprise a large diverse family of peptides that targets chloride, sodium, and potassium ion channels. The structure comprises an α-helix and an antiparallel triple-stranded β-sheet that is connected by two disulphide bridges. This fold is related to antimicrobial defensins and is found in arachnids, insects, mollusks and plants, and in tick salivary gland transcriptomes as defensins, but not as the CSαβ toxin scaffold [65,66]. The inhibitory cystine knot (ICK), disulphide-directed beta-hairpin (DDH), and single von Willebrand factor C-domain (SV-SVC) are a group of related peptide families. The ICK fold is considered to be prototypical for this group. Scorpions possess the classic ICK signature where cysteine three-four of the cysteine knot is adjacent and cysteine four-five have variable sized loops. In the cyclotide ICK signature, the three-four cysteine doublet is disrupted and cysteines four and five is separated by a single residue [67]. Both classic and cyclotide ICK motifs are considered to be ancient and ubiquitous, and have been recruited multiple times for various functions in diverse lineages [67]. Tick holocyclotoxin also present the ICK motif as a variant of the cyclotide ICK signature with the three-four cysteine doublet interrupted by a large loop, while two residues separate cysteine four-five [33]. Other tick protein family members that possess the ICK motif are the 5.3 kDa family [68], and they too exhibit the cyclotide ICK signature with the disruption of the three-four cysteine doublet and two residues that separate cysteine four-five (Figure 2).

### 3.4. Spider Toxins and Venoms

Spider venom is highly diverse and is composed of modulators of ion channels, transporters and G-protein coupled receptors (neurotoxins), cytolytic peptides, proteases, hyaluronidases, phospholipases, and sphingomyelinases [69]. The bulk of the venom is comprised of small peptides with molecular masses of 3–8.5 kDa that comprise cytolytic peptides and neurotoxins [69]. The majority of neurotoxins belong to the ICK family and are most possess the classic ICK signature [70]. While both scorpions and spiders possess the ICK fold and the ICK fold is considered to have evolved once or twice in eukaryotes (not in venomous lineages though), their evolution, expansion, and recruitment in scorpions and spiders are considered to be independent [67]. Other small cysteine-rich proteins, such as Kunitz-BPTI, are minimally present in spider and scorpion venoms, and are not major components as seen in tick saliva [66]. Similarly, enzymes in both spiders and scorpions include collagenases, hyaluronidases, phospholipase A2, and proteases [66,69]. These enzymatic activities also occur in ticks, but the information on these is limited so that no conclusion can be made regarding homology or common origin. Rather than being involved in direct toxicity, these activities are considered as spreading factors and are universal in all venomous organisms, but also those that feed from blood pools [53]. 

A large variety of tick salivary gland transcriptomes has been sequenced and all show the same distribution of salivary gland protein families. A limited number of protein families shows extensive gene duplication and include the basic tail secretory family (BTSP), Kunitz-BPTI, lipocalin, and metalloprotease families [36,46,53,71,72]. Expansion by gene duplication is a common feature of all blood-feeding arthropod sialomes and may be traced back to the ancestral protein repertoires of each lineage [36,46,72]. Each lineage also has its own distinct protein families that expanded. This is reflected in the protein family expansions that were observed in venomous arthropods as well [65,69]. In both cases, the evolution of new protein function by gene duplication from existing scaffolds seem to be easier than *de novo* evolution from many folds. Protein families that start to expand, tend to grow larger and this is not a function of a specific fold *per se*, but due to existing expansions [72]. As such, each lineage uses their unique families as scaffolds to evolve new functions. A measure of ancestral history of transcriptomes may therefore be found in the similarity of expanded families [36,46]. Soft and hard ticks share a common salivary gland transcriptome history, since they have the same protein family repertoire, and specifically the same expanded families, even though they have very few functional orthologs that are conserved between them [71].

### 3.5. Tick Toxins and Salivary Gland Proteins

Similarly, all of the triatomines share a common salivary gland transcriptome history and all mosquitoes share a common salivary gland transcriptome history [36]. It is also clear that comparison of salivary gland transcriptome compositions indicates that blood-feeding behaviour evolved at least 20× times independently in arthropods [53]. Similarly, it is clear that scorpions, spiders and ticks did not share a common transcriptome history with regard to their salivary and venom glands. On these grounds, it is unlikely that ticks share a venomous ancestral history with other venomous arachnids. Even so, all arthropods share the same common families that are found throughout non-venomous and venomous metazoans. These include apyrase, cystatin, defensin, hyaluronidase, ICK, Kunitz-BPTI, lipocalin, metalloprotease, and the many unmentioned families. It is therefore unsurprising that these families and functions are found in sialomes and venoms of blood-feeding and venomous arhropods, and is part of the recurring theme of convergent evolution [46]. In this regard, it should be noted that the ICK family thus far implicated in tick paralysis is limited to specific lineages (thus far *I. holocyclus*), and does not display the same expansions observed in other venomous arachnids, nor does it exhibit the same classic ICK signature, as extensively found in spiders and scorpions.

Heteropteran bugs can provide insight into the question on the evolution of a hematophagous lifestyle from a predominantly venomous predatory lifestyle. Heteropterans belong to the Hemiptera (true bugs), a large group (50,000–80,000 species) of mainly plant feeding insects that all possess piercing-sucking mouthparts to extract plant sap, which evolved ~300 MYA [73]. Heteropteran bugs evolved ~270 MYA, from this plant-feeding parasite to an ancestral lineage that is mainly predatory on other arthropods [73]. Within this lineage, the predatory Reduviidae lineage evolved ~178 MYA, while hematophagy evolved in the Triatominae only ~32 MYA [74]. The feeding mode of the predatory bugs encompass the paralysis of their prey using neurotoxins and subsequent liquefaction of the internal tissues, which the bug then ingests [75]. Recently, the venom transcriptomes from two predatory hemipterans, *Lethocerus distinctifemur* and *Pristhesancus plagipennis* have been described [76,77,78]. Both show the extensive presence of neurotoxins and digestive enzymes that exist as large multigene families. Conversely, the transcriptomes from blood-feeding triatomines do not show the presence of neurotoxins or digestive enzymes, but rather show the expansion of other protein families, such as the lipocalins and hemostatic system inhibitors [53]. It has been suggested that within the Triatominae, the *Rhodnius* and *Triatoma* lineages adapted independently to blood-feeding due to the few orthologs that exist between their transcriptomes [53]. However, their shared transcriptome protein family composition [53], as contrasted with the ancestral predatory venom transcriptomes, would suggest that blood-feeding did evolve once in the Triatominae. It also shows that hematophagous organisms that derived from a predatory venomous ancestor diverged from a venomous nature to evolve more benign functions, which is compatible with a parasitic lifestyle. This would suggest that the current salivary gland protein family composition of ticks would be completely different from a venomous or non-venomous ancestral lineage, as expected when compared to the genomes of other parasitiform mites, due to the requirements of adaptation to a blood-feeding lifestyle [36]. It would also suggest that most venomous traits would have been lost if ticks evolved from a predatory, to scavenger, to ecto-parasitic lifestyle.

We do not consider proteins that are involved in modulation of host hemostatic and immune defences as paralysis toxins, since the former do not elicit a paralysis phenotype. However, there is always the possibility that they can cause severe secondary effects, especially in non-natural hosts, for which no co-evolutionary adaption has taken place [40,61,79,80]. If ticks do not share a common venomous origin with other arachnids, major questions that remain is whether tick paralysis toxins are homologous and whether current knowledge explain paralysis phenomena. To address these questions, comparisons of data on tick paralysis from different lineages is necessary.

## 4. Phenotypic Commonalities and Differences between Paralysis Ticks

A comparative summary of commonalities and differences between major paralysis species is presented in Table 1. 

For the majority of venomous animals and insects, their venomous character is present in all strains/progeny of a species and they are capable of actively secreting toxic components in all life stages. For ticks, this generalization does not hold. Not all tick individuals from the same population or species may be able to cause paralysis, and not all life stages cause paralysis [60,61]. 

Even so, definite feeding phases are linked to the onset of paresis symptoms in all ticks that cause paralysis and coincide with the rapid feeding phase, generally 3–7 days into feeding [6]. Paresis symptoms start as an ascending flaccid tetraplegia, because it affects the nervous system of the host [6]. In soft ticks, only larvae cause paralysis since they feed slowly for several days, while nymphs and adults feed fast within minutes to hours. The only exception to this is *Ornithodoros lahorensis*, where the slow-feeding third stage nymphs cause paralysis in sheep [5].

In hard ticks, different feeding stages from different tick species cause paralysis. In *I. holocyclus*, females mainly cause paralysis, although nymphs have also been reported, but not larvae [81]. Nymphs of *I. rubicundus* mainly cause paralysis, although females have been implicated in the field [39,82]. In the case of *Dermacentor*, females of *D. andersoni*, nymphs of *D. rhinocerinus* and females of *D. variabilis* cause paralysis [83,84,85]. For *R. evertsi evertsi,* females cause paralysis within a very narrow mass range of 15–21 mg body weight [6].

The number of ticks that can cause paralysis markedly differ between species. For *D. rhinocerinus*, 50 nymphs are capable of paralysis in a rabbit [84], while at least 200 nymphs were needed for paralysis of a lamb by *I. rubicundus* [82]. On the other hand, a single female from *D. andersoni* or *I. holocyclus* has been reported to be able to kill a cow, dog, or even a human [86,87]. Three hundred larvae from *A. walkerae* caused full paralysis in chickens [88]. Host size and dosage are obviously linked, but toxin potency and mechanism must also play role in these observations.

Not all vertebrates are affected by tick paralysis. *Argas walkerae* only produces paralysis in chickens, but also probably target game birds in the wild [6]. Animals that are seriously affected by the bite of *I. holocyclus* are livestock i.e., sheep, goats, horses, pigs, chickens, and ducks. Companion animals affected are cats and dogs, but they also frequent humans [9]. *Ixodes rubicundus* can paralyze sheep, goats, antelope, cattle, dogs, and humans [39]. *Rhipicephalus evertsi evertsi* cause paralysis in sheep only and do not cause paralysis in cattle, gerbils, guinea pigs, hamsters, mice, multimammate mice, rabbits, or rats [6]. The paralysis toxin from *D. andersoni* is also of interest, since it primarily affects humans, cattle, dogs, horses, and sheep. In the laboratory coyote, guinea pigs, hamsters, marmots, monkeys, mule deer, pack rats, and squirrels could be paralyzed. Conversely, cats, mice, pheasant, porcupines, rabbits, and wild muskrat did not show paralysis [86]. Reasons for host susceptibility is unknown at this time.

The paralysis phenotype (ascending flaccid paralysis) is similar for all tick species, although some differences exist, but data are not clear enough to make conclusions regarding the homology or conservation of mechanism. In *A. walkerae*, the peripheral nervous system, especially fast conducting nerve fibers, is affected, leading to a decreased motor conduction velocity of the median-ulnar and sciatic nerves [6,89,90]. This was interpreted as blockage of increasing numbers of sciatic nerve fibers that may be described as a motor polyneuropathy that does not significantly affect afferent paths. The efferent nerve fibers of the breathing muscles are also affected but not those of the heart, resulting in eventual cessation of breathing. Acetylcholine release at the neuromuscular junction and receptor sensitivity at the myoneural synapse is affected [6,91]. Larval extracts inhibited the potassium stimulated (calcium-dependent) and veratridine-stimulated (external calcium-independent) release of [^3^H]glycine from crude rat brain synaptosomes [91]. This was considered evidence that synthesis or release of acetylcholine is inhibited.

In *D. andersoni*, peripheral nerves are targeted with the impairment of impulse propagation at the myoneural or spinal cord synapses with little effect on the afferent pathways. This impairment may be due to the inhibition of acetylcholine release or acetylcholine destruction by an anti-cholinesterase. The acetylcholine receptor does not seem to be inhibited, but the synthesis or release at the nerve terminals may be affected [5]. The afferent nerves are not affected, but only the motor fibers. The site of action is probably at the terminal motor fibers on the neuromuscular junctions. Paralysis exhibits as a motor polyneuropathy of the peripheral nerves with limited participation of the afferent pathways [6].

In *R. evertsi evertsi*, paralysis exhibits as a motor polyneuropathy of the peripheral nervous system. Primarily, the slower conducting nerve fibers are blocked. The efferent fibers of the breathing muscles are also affected until paralysis leads to respiratory failure. The effect on acetylcholine release or inhibition has not been investigated.

In *I. holocyclus*, peripheral nerves and the cerebral cortex are not affected, while the motor anterior horn neurons and cranial nerve cells seem to be affected. A direct temperature-sensitive action on the excitation-secretion mechanism of the myoneural synapse was indicated [92]. Acetylcholine release was inhibited above 30 °C, which was possibly due to the targeting of processes between depolarization of the terminal membrane and release. Minimal effect on miniature end-plate potentials of a mouse extensor *digitorum longus* nerve-muscle preparation suggested that neither calcium-independent release of vesicles nor post synaptic acetylcholine receptors were targeted by synthesized holocyclotoxins [34]. However, end plate potentials were reduced in a calcium-dependent manner, suggesting a presynaptic mechanism, which was possible between depolarization and the calcium dependent release of vesicles. This would suggest direct or indirect targeting of the voltage gated calcium channels [34]. Targeting of calcium channels are found in spiders and scorpions by ICK peptides. However, in spiders and scorpions, an extensive range of inhibitors of presynaptic potassium and sodium channels are also found, rendering the presence of a single family that is responsible for paralysis in ticks intriguing [40,65,69,93].

All differences may be related to toxin dosage, number of toxins in the saliva, differential expression of toxins in different life stages or time of feeding, feeding site, host body mass, toxin target, or toxin affinity for its receptor. It may also be due to different types of toxins and whether proteins involved in hemostatic and immune modulation of host defenses can have synergistic effects on paralysis. To ultimately draw conclusions regarding the evolution and origin of tick toxins, they need to be characterized at molecular level. The subsequent Sections aim to illuminate our knowledge regarding the molecular nature of tick paralysis toxins, strategies to characterize them, the challenges faced to attain this and what the implication of these strategies are for our understanding of paralysis phenomena.

## 5. Molecular Data on Tick Toxins

If tick paralysis toxins evolved from a common ancestral toxin gene, then it may be expected that they may share common characteristics since they may target the same receptor and have the same function. However, characterization of toxins from different species has indicated many differences and discrepancies.

In *I. holocyclus*, the isolation and purification of the holocyclotoxin resulted in varying molecular sizes for purified fractions from salivary gland extracts. This was ascribed to variable resolution in electrophoresis protocols. A different, probably more accurate explanation for this, were proposed by Malik [7], that saliva was comprised of different toxic components; the paralysis causing holocyclotoxin, as well as other lethal but not paralysis causing toxins [7]. Initially, the molecular weight for holocylotoxin was reported as 40–80 kDa [32]. Subsequently, the toxic components were attributed to three polypeptides, which were known as holocyclotoxin 1, 2, and 3 (HT1, HT2, and HT3), with an estimated molecular mass of 5 kDa each [94]. It should be noted that different purification schemes used different bioassays to purify toxins (see Sections below). Another toxic lethal fraction of ~20 kDa has been associated with cardiovascular failure, but has not been characterized on molecular level [32]. Recently, sequencing of the transcriptome of *I. holocycus* identified 16 additional members of the holocyclotoxin family [35]. This family only has homologs within *Ixodes* [68], and as such represent an orphan family. Chemical synthesis and testing of HT1, HT2, HT3, and HT4 indicated that HT4 was more potent than the original isolated toxins, while HT12 showed similar activities to HT3 [34,35]. It may therefore be possible that the holocyclotoxins have a range of toxicities and physical properties that may explain the previous observed differences.

For *A. walkerae*, different purification methods that were used for toxin isolation resulted in different sized toxic fractions. Viljoen [88] determined the toxin to be an oligomer of 18, 32, and 60 kDa proteins that was isolated using a bioassay and chromatography [88]. Maritz, C. et al. on the other hand isolated an 11 kDa fraction using a monoclonal antibody that was directed against the toxin of *R. evertsi evertsi* [95]. No new work has yet resolved these differences. 

The *R. evertsi evertsi* toxin was purified using a bioassay and chromatography to yield a pure product of 68 and 74 kDa on size exclusion chromatography and sodium dodecyl sulfate polyacrylamide gel electrophoresis (SDS-PAGE) respectively [96]. A monoclonal antibody that was raised against the toxic phase detected three immunogenic bands at 23, 46, and 69 kDa [97]. The toxin was suggested to be associated with the 69 kDa protein, while the lower masses was suggested to be degradation products. Crause, J.C. et al. [59] suggested the function of the toxin of *R. evertsi evertsi* to be a regulator of protein synthesis based on the localization staining to chromatin in the nuclei. Immunity of recovered animals were limited [12,98].

No molecular characterization data exists for *I. rubicundus* or *D. andersoni*.

In comparison: holocyclotoxins are between 40–60 kDa [99] with a toxic fractions of 5 kDa [33,94], 68 kDa for the toxin of *R. evertsi evertsi* [96], and 18–60 kDa for *A. walkerae* [88], with a potential low molecular mass toxin of 11 kDa [95]. As yet, it is not clear whether the molecular differences observed are artifacts and whether all of the toxins actually have molecular masses that approximate 5 kDa, and have ICK folds. With the data at hand, it remains difficult to conclude that the toxins are homologs that share a common evolutionary origin. The question therefore turns to how we might characterize these toxins and derive at answers to their origin.

## 6. Strategies to Identify and Characterize Tick Paralysis Toxins

### 6.1. Purification of Paralysis Toxins Using Paralysis As Assay

The gold standard for the monitoring of paralysis inducing capability during the purification of paralysis toxins is a direct assay for paralysis in the natural target host or any other host that displays a paralysis phenotype. However, this has proven to be extremely difficult for the majority of paralysis toxins since paralysis cannot always be induced by injection of salivary gland or whole body extracts [96]. This was the case for *R. evertsi evertsi* [96] and *D. andersoni* [5]. Extracts from other paralysis inducing ticks that have never been tested include *D. rhinocerinus*, *D. variabilis*, and *I. rubicundus*.

The only toxins to date that gave appreciable paralysis results were paralysis toxins from *A. walkerae* and *I. holocyclus*. In the case of *I. holocyclus*, neonatal mice and dogs could be paralyzed using salivary gland extract [100,101]. Ion exchange chromatography followed by paralysis monitoring in mice and dogs resulted in the first purification of a paralysis fraction from *I. holocyclus* [102]. The ability to induce paralysis from purified fractions allowed extensive attempts to purify the toxin [94,99,102,103,104]. These studies identified various proteins with molecular masses ranging from 40–80 kDa, as well as 5 kDa. Whole body extracts of replete larvae from *A. walkerae* induced total paralysis in one-day-old chickens and fractionation using size exclusion chromatography, followed by ion exchange chromatography, resulted in a purified fraction with two major components of 32 kDa and 60 kDa on SDS-PAGE [88]. These studies indicated that although not possible for all paralysis tick species, it is possible to purify specific proteins that cause paralysis. Also extremely important, and bound to impact on most of our understanding of paralysis, is quantification of toxin in terms of yield and specific activity. It will also be important to know whether purified toxins recapitulate the complexity of paralysis induced by feeding ticks. The major issue would be whether we are missing paralysis toxins in any alternative scheme, as detailed below.

### 6.2. Purification of Paralysis Toxins Based on Bioassays and Molecular Tools

In line with the 3R’s of modern animal ethics (Replacement, Reduction, and Refinement), a number of alternative bioassays and methods have been used over the years to characterize tick paralysis toxins and to use during their purification. 

A first degree removed from a direct paralysis assay is the use of whole organ preparations. For *R. evertsi evertsi*, a frog leg sciatic nerve-muscle preparation was used to monitor muscle paralysis [96,105]. Chromatographic purification and monitoring with this bioassay resulted in the purification of an 86 kDa protein. The effect on a frog nerve-muscle preparation is intriguing, since it raises the question of whether the same target and/or toxins are involved in sheep and frogs, since few other mammals are affected by feeding *R. evertsi evertsi* ticks. Organ systems that were investigated for *D. andersoni* included crayfish stretch receptor muscles, frog nerve-muscle, rat diaphragm, and lumbrical muscle preparations, of which none gave answers [86]. A rat phrenic nerve-hemidiaphragm preparation from paralyzed mice was successfully used to show that the toxin exhibit temperature dependent paralysis [92]. Since mice are hosts that can be paralyzed, this model may be particularly useful even if it may only work from already paralyzed animals. The use of organ preparations presume that the system of choice resemble the physiological state that was found in the natural host and do raise the question of why any organ preparation from any vertebrate host is not successful, given the general conservation of the peripheral neural system and especially the presynaptic synapse.

**Figure 3:**
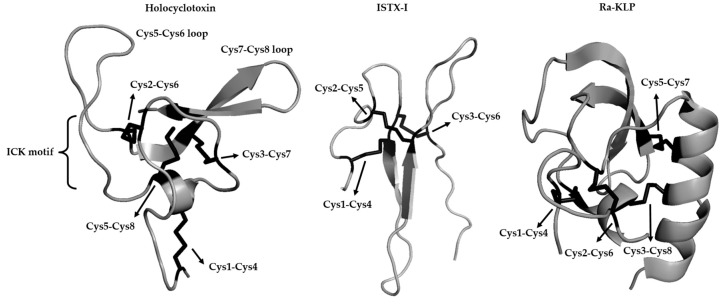
Structures of ion channel inhibitors from ticks. Indicated are holocyclotoxin from *Ixodes holocyclus* displaying the ICK motif, *Ixodes scapularis* neurotoxin I (ISTX-I) that does not possess a ICK motif and the activator of Ca^2+^ activated potassium (K^+^) channels that were identified in *R. appendiculatus*, *R. appendiculatus* (Ra-KLP) that belongs to the Kunitz-BPTI fold.

A second degree removal would be the use of organ homogenates. In the case of *I. holocyclus*, the holocyclotoxins (HT1, HT2, and HT3) with a molecular mass of ~5kDa have been purified using temperature dependent binding to rat brain synaptosomes, which presumably model the presynaptic synapse [94]. Cloning and sequencing of HT1 generated a secretory protein of 5.9 kDa with a pI of 8.86 and eight cysteines in the mature protein [81]. High homology to scorpion neurotoxins was indicated, but recently the structure of a chemical synthesized peptide indicated that although the overall fold possesses the cystine knot motif, the tertiary structure is novel (Figure 3) [33]. The authors suggested that although the cystine knot motif is common in spiders and scorpions, it probably evolved independently in tick toxins. Chemically synthesized HT1, HT3, and HT12 (a homolog) indicated the targeting of voltage gated calcium channels [34]. It should be noted that very high concentrations (IC50~5–12 mM) were necessary for inhibition. This would not resemble any physiological effect since these concentrations would not be present in saliva, although 20 µL crude saliva (14 µg total protein) could elicit a similar response. If it was assumed that all crude salivary protein was toxin, then this would have given an IC50 of 140 µM, which is ~35–85 fold less than the chemically synthesized peptides. This would suggest that the peptides were incorrectly folded or that other toxins exist in the saliva that may be more potent. The more recent additions to the HT family, as identified using *I. holocyclus* transcriptome sequencing and chemical synthesis, confirmed that HT1, HT2, and HT3 bound to synaptosomes [35]. Only partial paralysis could be obtained in 5 g neonatal mice using 30 µg of HT1, HT2, or HT3, although when combined (90 µg), they produced complete paralysis. HT4, on the other hand, produced complete paralysis at 30 µg. It was suggested that paralysis is mediated by a whole family of neurotoxins rather than only one. This raises an important point that the use of bioassays to replace paralysis may result in the purification of only subsets of potential toxins. In this case a homology/transcriptome approach allowed for the discovery of additional homologs (see below).

A third degree of separation constitutes the functional characterization of molecules that target known receptors of neurotoxins, such as ion channels. As such, an activator of Ca^2+^ activated potassium (K^+^) (maxiK) channels were identified in *R. appendiculatus* (Ra-KLP), which belong to the Kunitz-BPTI family [106], that is not evolutionary related to the ICK fold (Figure 3). Kunitz-BPTI members that inhibit potassium channels (Kv channels involved at the presynaptic synapse) has been found in dendrotoxins (snakes) and toxins from sea anemones. The function of Ra-KLP is not clear, but it may be involved in enhancing the blood flow at the feeding site, although the concentrations needed for activity is not physiologically significant [106]. The fact that *R. appendiculatus* is also not a typical paralysis-inducing species make the assessment of this functionality difficult, but it raises the point that ion channel inhibition would not necessarily result in paralysis.

More recently, a sodium channel inhibitor (ISTX-I) has been characterized from the non-paralysis inducing species *I. scapularis* [107]. The authors explicitly refer to ISTX-I as a neurotoxin. It has a novel fold that is not related to any known fold, including the inhibitory cysteine knot (ICK) fold (Figure 3), even if it superficially has the same disulphide bond pattern (C1–C4, C2–C5, C3–C6), but does not form the cystine knot. The only other hit retrieved using PSI-BLAST analysis is a homolog from *I. pacificus* (AAT92145), which is another non-paralysis inducing tick species. It was therefore interesting that the authors compared ISTX-I to spider toxins that possess the ICK motif that targets sodium channels and scorpion toxins with ICK motifs that are related to defensins and potassium channel blockers without ICK motifs. It should be noted that the ICK motif is a structural scaffold that is derived independently from various lineages and has been incorporated into many different folds [67]. A phylogenetic analysis was performed using these non-homologous sequences that yielded a non-informative tree from which no substantial conclusion may be derived since these proteins are not evolutionary related [107]. ISTX-I had a IC50 of ~1 μM for the Nav1.7 sodium channel, which is 5–100-fold higher than normally observed for sodium channel blockers [70]. As such, ISTX-I would probably not be secreted at physiologically relevant concentrations that would impact on host biology, but has been suggested to play a role in modulation of pain. Of interest, is that *I. scapularis* possess a potent kininase activity in its saliva that targets bradykinin (a modulator of pain), which probably function in the role of pain modulator [108,109]. As a final comment on this Section, it may be possible to discover many channel inhibitory or modulatory activities in ticks that cannot be linked with a paralysis or toxicoses inducing phenotype. It again underscores the question of what constitutes a toxin and whether biochemical activity constitutes the proof of a venomous nature.

A fourth degree of separation constitutes the use of molecular tools, such as antibodies to monitor paralysis. In the case of *R. evertsi evertsi*, a monoclonal antibody was preferentially generated against the paralysis phase of feeding, by immunosuppression of anti-tick responses to any proteins from the previous feeding stages [58]. The monoclonal antibody detected proteins at 23, 46, and 69 kDa, respectively [58], and could be localized to nuclei and secretory granules of b-type salivary cells [59]. Significantly, the monoclonal antibody was capable of inhibiting paralysis induced by the salivary gland extract in the frog nerve-muscle preparation [97], suggesting that the antibody targets the major contributor to paralysis in this tick species. Even so, the use of an antibody as detection tool, would allow for the detection of closely related homologs, but might miss non-related toxins.

A fifth degree of separation constitutes the use of a monoclonal antibody that detects a paralysis toxin from one species to detect toxins from other species. Since antibodies only detect epitopes of ~8–15 amino acid residues, the assumption would be that these epitopes also exist in the paralysis toxins from other tick species. Using this approach, significant cross-reactivity was observed with paralysis-inducing tick species and strains, such as *A. walkerae*, *I. rubicundus*, and *R. evertsi evertsi*, but not *Hyalomma rufipes*, *R. appendiculatus*, *R. decoloratus*, or a non-paralysis inducing strain of *R. evertsi evertsi* [97]. The monoclonal antibody also protected one-day-old chicks against paralysis induced by *A. walkerae*. This would imply that paralysis toxins from tick lineages with distant genetic relationships (*Argas*, *Ixodes*, *Rhipicephalus*) share common epitopes or even homologous relationships. Common epitopes could arise through convergent evolution if these target the same molecular target, with the implication that the monoclonal antibody may target the active site residues of the toxin. The null hypotheses that all ticks share a common ancestry, and therefore common homologs would suggest that cross-reactivity is due to homology.

A sixth degree of separation considers the case of homology. It has been shown that protein structures are more conserved than DNA sequences amongst homologues and the resulting sequence similarity usually implies structural similarity [110], therefore evolutionary related proteins should have similar sequences and protein structures. These proteins can be related to a protein super family for which common ancestry can be inferred based on their structural alignment. Since only a single protein family, the holocyclotoxins, has been causally associated with paralysis, their possible homologs can be searched for in the sequence databases. No homology could be detected using BLAST or structural similarity searches [33]. An up to date PSI-BLAST search (January 2018) only retrieved other family members of the holocyclotoxin family in *I. holocyclus*. Alignment of these members shows very little sequence conservation beyond their disulphide bond pattern. Database searches include mostly non-paralysis species for which genomic or transcriptomic data is available for >40 transcriptomes, which is expanding rapidly [36]. The argument may be raised that holocyclotoxin, given its nature as a small peptide could be easily missed, or that the current salivary gland transcriptome databases are incomplete. A relationship to the 5.3 kDa family from *I. scapularis* was indicated previously [68]. Comparison of the 5.3 kDa family with the holocyclotoxins indicates differences, which include six cysteines (three disulphide bonds) for the 5.3 kDa family and eight cysteines (four disulphide bonds) for the holocyclotoxins. The disulphide bond pattern for three disulphide bonds are conserved, while the last is lacking in the 5.3 kDa family (Figure 2). While uneven disulphide bonds are uncommon in protein families, it does occur as seen for example in some members of the Kunitz-BPTI family and even the ICK fold [67,106,111]. In this case the conserved disulphide bonds form the inhibitor cystine-knot motif [33], suggesting that the 5.3 kDa family display this motif as well. The other significant difference between the holocyclotoxins and the 5.3 kDa family are the extended loop between cysteine 5 and 6, suggesting that this loop may play an important role in neurotoxicity (Figure 2 and Figure 3). Even so, few residues are conserved in this loop. The other region that may potentially be involved in neurotoxicity concerns the loop between cysteine 7 and 8 (Figure 2 and Figure 3).

A seventh degree of separation related to the direct homology searches of known paralysis toxins from ticks, is to ask whether ticks have other, as yet undiscovered, toxins that are homologous to other known venomous arthropods. This may take the form of single BLAST searches of known paralysis toxin sequences or to ask the broader question of how conserved salivary gland transcriptomes are compared to venom transcriptomes. This may identify distant homologs, but also address the question on the ancestral conservation of venomous traits in arachnids. Given the number of tick transcriptomes that were sequenced, it may be assumed that the majority of tick protein family folds have been discovered [36]. As indicated in Section 3.3, Section 3.4 and Section 3.5, tick transcriptomes only resemble spider or scorpion toxins in the broadest sense. The major expanded families are not conserved and indicate definite independent trajectories in the evolution of tick and venomous arachnid transcriptomes. Indeed, even ticks and other parasitiform mites do not share extensive orthologs that can be traced to tick sialomes [36]. However, until well described and annotated tick salivary gland transcriptomes for all tick species that are implicated in paralysis have been generated, no conclusion regarding affinities of potential paralysis toxins from ticks and venomous arachnids can be made. In this regard, a comparative analysis of transcriptome data between paralysis causing tick species is necessary to identify orthologs. It might also shed some light on why certain strains of the same species are capable of causing paralysis and others not. By sequencing the transcriptomes of paralysis and non-paralysis inducing ticks the search for inter-transcriptome active elements, toxins or orthologs thereof, will shed some light on the evolutionary adaption of paralysis inducing ticks to their hematophagous life and advance our understanding of general tick salivary gland biology. The analysis of the transcriptome and proteome can collectively give a global view of expression patterns, as any protein uniquely expressed during the paralysis phase, might be the potential toxin or be capable of contributing to the “cocktail” that is injected during feeding, causing toxicoses or paralysis. Once the transcriptome has been catalogued against the proteome, comparative bioinformatics can aid in the identification of possible orthologs in the Arachnida, enabling prediction of functions based on homology and functional motif analysis. Even if the transcriptome describes all potential genes transcribed at different time points during feeding, the only way to validate it is with a detailed proteomic analysis.

An eight degree of separation is the use of bioinformatics to predict toxin properties in protein folds with known neurotoxic associations. The rationale would be that toxins exhibit motifs, patterns, or signatures that are associated with the molecular mechanisms of paralysis function [112]. This approach has a time honored history with known motifs that are associated with various functions [113,114]. In the case of toxins, numerous approaches have been implemented to derive generalized descriptions of toxin functionality [40,41,112,115]. These may not necessarily depend on homology, but rather aim to treat toxin folds as scaffolds where recurring themes are presented. As such, conserved residues may provide clues to functionality [112]. However, in most cysteine-rich peptide families, the only conserved residues are the cysteines that are involved in disulphide bonds, as exhibited for the ICK fold [67]. Specific residues may also be conserved, as for example in the Kunitz-BPTI fold, where the occupation of the P1 position of the substrate binding presenting loop by a basic amino acid, such as arginine or lysine, generally indicate the targeting of trypsin-like serine proteases [116]. However, this is context dependent and exceptions to the norm may occur, as is the case for the savignygrins, where the P1 position is an arginine, but serine proteases are not targeted, since the arginine forms part of another well-known motif: the RGD motif that is involved in integrin α_IIb_β_3_ interaction [117]. Other approaches include the use of surface charge distribution, with the rationale that positively charged surfaces may be prone to target ion channels [40,41]. In terms of the prediction of ion channel modulators for ticks, conserved residues [118] and complex machine learning algorithms that incorporate an array of variables to analyze sequence composition [115] have been used. While such approaches can certainly yield candidates for analysis, the final arbiter should remain empirical confirmation within a physiologically significant context.

The degrees of separation that are discussed here shows a distinct and increasing dissociation from the gold standard paralysis inducing phenotype to a somewhat gray area where toxicity is defined in terms of homology, structural fold, or family similarity, or even based on conserved sequence motifs. This is a significant deviation from a reductionist approach that aims at the identification of discrete units of toxicity, which are responsible for the paralysis phenotype, using the hypothesis that specific proteins are responsible for paralysis. Systems biology approaches may be useful in the analysis of tick salivary gland complexity, but the majority of functions that are involved in the modulation of tick hemostatic and immune defenses can still be reduced to single proteins and analyzed, according to defined functions [36]. For tick paralysis, this reductionist approach should also be the Holy Grail, since this holds promise for development of defined anti-paralysis vaccines.

## 7. Conclusions

Evolutionary considerations that are based on systematic relationships among arachnids, morphological adaptations to specific venomous or blood-feeding lifestyles, venom and salivary gland transcriptome compositions, and the molecular properties of known paralysis toxins do not support a common venomous origin in the Arachnida, nor a classification as being venomous for ticks. The homology of tick paralysis toxins also needs to be confirmed, since even this is uncertain at this time. How we define a toxin is also important and crucial to the endeavor to identify tick paralysis toxins. A final caveat should be stated. With enough degrees of separation from the gold standard paralysis phenotype, almost any tick-derived protein may be called a toxin. As Paracelsus indicated: the dose makes the poison. In ticks, this is determined by the amount of protein that can be secreted by a tick during a feeding session that would induce a paralysis or toxicoses inducing phenotype. Rigorous and critical analysis will indicate that toxins need to be abundant and highly potent (high affinity and low LD50) to fulfill these criteria. Ticks need to be observed in their natural surroundings to determine whether they consistently produce paralysis or other toxicoses. If this does not occur on a repetitive basis, toxicoses or paralysis may be an incidental phenomenon that is not associated with tick feeding. If toxins can cause paralysis in whole animals by ticks, they should be detectable using the empirical methods described in Section 6.

The current review focused on tick-derived salivary gland molecules that may be involved in tick paralysis. However, as indicated previously [8], toxins may also be non-tick-derived, originating from symbionts or even modified host proteins. In these cases, the origin of toxins would probably still not derive from a venomous ancestral arachnid, but would be a by-product of symbiosis or blood meal digestion. Non-proteinaceous monoaminergic components in venoms, such as dopamine, epinephrine, histamine, norepinephrine, octopamine, serotonin, and tyramine, may be used to cause immobilizing hyperexcitation [119]. However, instead of secreting such biogenic amines, ticks, and other hematophagous arthropods produce potent scavengers of these molecules, since they play important roles in vertebrate host inflammatory responses [120]. While non-tick-derived and non-proteinaceous origins of tick paralysis toxins should always be considered and excluded, our current data suggest that tick paralysis toxins are salivary gland derived proteins, which are possibly involved in benign feeding functions.

Are ticks venomous? This may depend on your philosophical outlook. We present, as final consideration, two definitions modified from Fry, B.G. et al., 2009 [66] to distinguish venomous organisms from their more benign counterparts. Venomous organisms secrete venom that is: “a secretion, produced in a specialized venom gland in one animal and delivered rapidly (in seconds) to a target animal through the infliction of a wound (regardless of how tiny it may be), which contains molecules that disrupt the normal physiological or biochemical processes in the victim so as to facilitate defense or predation by the producing animal. This pharmacological cocktail aims to induce pain, deter, immobilize or kill the aggressor or prey”. On the other hand, hematophagous parasites secrete a biopharmacopeae that is: “a secretion, produced in a salivary gland in one animal and delivered over minutes or days to a target animal through the infliction of a wound (regardless of how tiny it may be), which contains molecules that disrupt normal host defenses (hemostasis, inflammation and immune responses), so as to facilitate efficient feeding to obtain a blood meal. This pharmacological cocktail aims to suppress pain and inflammation and disrupt hemostasis to an extent that would allow sustained parasitism and survival”. As was seen for the conversion of assassin bugs from lethal predators to secretive phlebotomists, the hematophagous organism needs to tailor their responses in order to cause minimal damage to the host, while tricking it into the surrender of its most precious cargo, blood. In this sense, they truly are pharmacologists [121]. Venomous organisms, on the other hand, are smart bomb operators, with the sole purpose to intentionally deter or to kill their enemies or prey. The major question remains, whether tick paralysis and toxicoses are functional adaptations of ticks, or an accidental by-product in the vertebrate hosts affected.

## Figures and Tables

**Table 1:** Characteristics of known tick paralysis toxins.

Characteristic	*I. holocyclus*	*R. evertsi evertsi*	*A. walkerae*	*D. andersoni*
Life stage that cause paralysis	Nymphs and adults	Adults	Larvae	Adults
Mechanism of toxin	Inhibits synaptic vesicle (acetylcholine) release when binding to the synaptosomes at neuromuscular junction	Impair the conduction of impulses along the peripheral nerve fibers (nodes of Ranvier)	Inhibits Ca^2+^ dependent synaptic vesicle release and desensitizing its receptor	Motor polyneuropahty with limited participation of the afferent pathways
Size	40–80 kDa; HT-1 = 5kDa	68–70 kDa 74 kDa	11 kDa/range of 11–115 kDa 80–100 kDa/32 and 60 kDa	37–43 kDa
Recovery after tick removal	Prolonged (Days to weeks) with initial deterioration of host’s condition	Within hours to two days	Within hours	Within hours
Antiserum therapy	Useful in early stage of paralysis	None available	None available	None available
Immunity	Full	Limited	Partial	Dose dependent immunity
Isoelectric point	8.86 4.5–5	6	4.5	Unknown
Protease digestion	Resistant	Inactivate toxin	Unknown	Unknown
